# *C2* rs547154 polymorphism and polypoidal choroidal vasculopathy susceptibility: a meta-analysis

**DOI:** 10.1038/srep08709

**Published:** 2015-03-03

**Authors:** Xue Chen, Xiaoli Kang, Kanxing Zhao, Chen Zhao

**Affiliations:** 1 Department of Ophthalmology, The First Affiliated Hospital of Nanjing Medical University, State Key Laboratory of Reproductive Medicine, Nanjing, China; 2 Department of Ophthalmology, Xinhua Hospital, Shanghai Jiao Tong University School of Medicine, Shanghai, China; 3 Tianjin Medical University, Tianjin Eye Hospital, Tianjin Key Laboratory of Ophthalmology and Visual Science, Tianjin, China

## Abstract

Previous studies have indicated the association between *C2* rs547154 polymorphism and polypoidal choroidal vasculopathy (PCV) risk, while the results are controversial and inconsistent. Herein, we perform a meta-analysis to gain a precise estimation of the association using 5 eligible studies involving 4076 subjects, of which 1220 were PCV cases, 1073 were age-related macular degeneration (AMD) cases and 1783 were controls. Allelic frequencies of *C2* rs547154 polymorphism between PCV and AMD were also compared. Both crude and adjusted odds ratios (OR) with their 95% confidence interval (CI) were included to assess the strength of the association. The pooled OR in random-effect model for allele T versus G was 0.64 (95% CI, 0.52–0.80; *p* < 0.0001), for genotype TG versus GG was 0.65 (95% CI, 0.52–0.83; *p*, 0.0004), and for genotype TT + TG versus GG was 0.64 (95% CI, 0.51–0.80; *p*, 0.0002). No difference in allelic frequency was observed between PCV and AMD (OR, 0.86; 95% CI, 0.64–1.16; *p*, 0.32). Sensitivity analysis proved the robustness of our data. No significant ethnic divergence was suggested by subgroup analysis, and no publication bias was detected via Egger's test. In conclusion, our data indicate that *C2* rs547154 polymorphism plays a protective role in the development of PCV.

Polypoidal choroidal vasculopathy (PCV) is a hemorrhagic and exudative maculopathy characterized by hyalinization and peripapillary, macular or peripheral sub-retinal pigment epithelium (RPE) polypoidal dilatations of branching choroidal vascular networks in indocyanine green angiography (ICGA) presentations^1^,^2^,^3^, which may subsequently lead to detachments of RPE, or even neurosensory retina^2^,^4^,^5^,^6^,^7^. Due to the many shared clinical hallmarks with age-related macular degeneration (AMD), including recurrent subretinal hemorrhage, exudation, serous and hemorrhagic RPE detachment, and vitreous hemorrhage, PCV remains unrecognized for a long period until its first identification by Yannuzzi et al in 1990^2^. Recent studies indicate more unique manifestations in PCV that distinguished PCV from AMD, suggesting that PCV is a clinical entity separated from AMD^1^,^8^,^9^.

The pathogenesis of PCV remains elusive, while it is widely accepted that both genetic and environmental factors play important roles in the disease course of PCV. Considering the clinical similarities between AMD and PCV, researchers aim to find out whether AMD and PCV share common pathogenic pathways. Previous meta-analyses have reported that age-related maculopathy susceptibility 2 (*ARMS2*; MIM: 611313) rs10490924 and complement factor H (*CFH*; MIM: 134370) rs800292 polymorphisms, variants implicated in AMD etiology, might also increase PCV risk^10^,^11^. The complement component 2 (*C2*; MIM: 613927) rs547154 variant is a G to T substitution located on chromosome 6p21, which shows protective effect against AMD^12^,^13^. This variant has been widely investigated for its association with PCV^14^,^15^,^16^,^17^,^18^, while the results are controversial and the sample size in each study is limited. Therefore, we performed this meta-analysis to assess the relationship between the *C2* variant and PCV susceptibility.

## Results

### Literature

**Figure 1:**
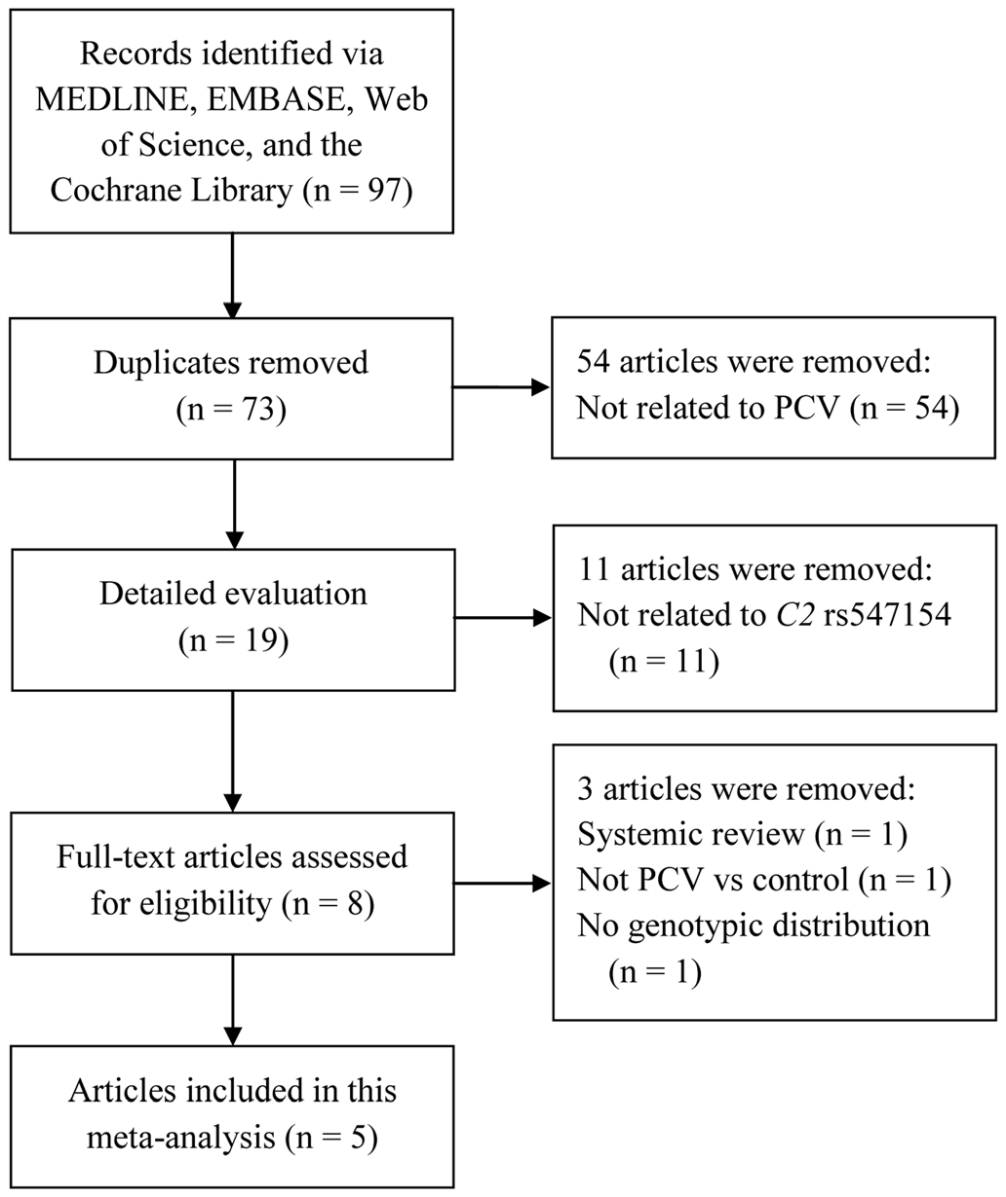
Flow chart depicting the screening process for inclusion in the meta-analysis.

The initial literature search yielded 97 articles relevant to the search terms. Five articles, published from 2008 to 2014, embodying 4076 subjects (1220 PCV cases, 1073 AMD cases and 1783 controls) were finally included for the meta-analysis^14^,^15^,^16^,^17^,^18^. Flow chart of literature screening and review was shown in Figure 1. All five were case-control studies in English with their characteristics listed in Table 1. The average ages ranged from 63.80 to 73.00 years in the PCV case group, from 73.60 to 75.59 years in the AMD case group, and from 48.22 to 72.90 years in the control group, while the gender ratios (male/female) in the three groups varied from 1.39 (32/23) to 3.39 (105/31) for PCV cases, 2.64 (330/125) to 2.97 (187/63) for AMD cases, and 0.67 (110/164) to 1.51 (110/73) for controls (Table 1). Of the 5 studies, 3 were in Japan^15^,^17^,^18^, 1 was in Singapore^14^, and another one was in USA^16^. Three studies used TaqMan for genotyping^15^,^17^,^18^ and one used Sanger sequencing^14^, whereas both polymerase chain reaction-restriction fragment length polymorphism (PCR-RFLP) and TaqMan were applied in the study performed by Lima et al^16^.

### Meta-analysis

Allelic and genotypic distributions for the *C2* rs547154 variant from each individual study are presented in Table 2. The genotypic distribution in the control group was consistent with Hardy-Weinberg equilibrium (HWE) in each study. The adjusted odds ratios (OR) with their 95% confidence interval (CI) and the corresponding adjusted variables were provided by two studies and were listed in Table 2^17^,^18^. Quality assessments of all included studies with the Newcastle-Ottawa Scale were included in Table S1.

**Figure 2:**
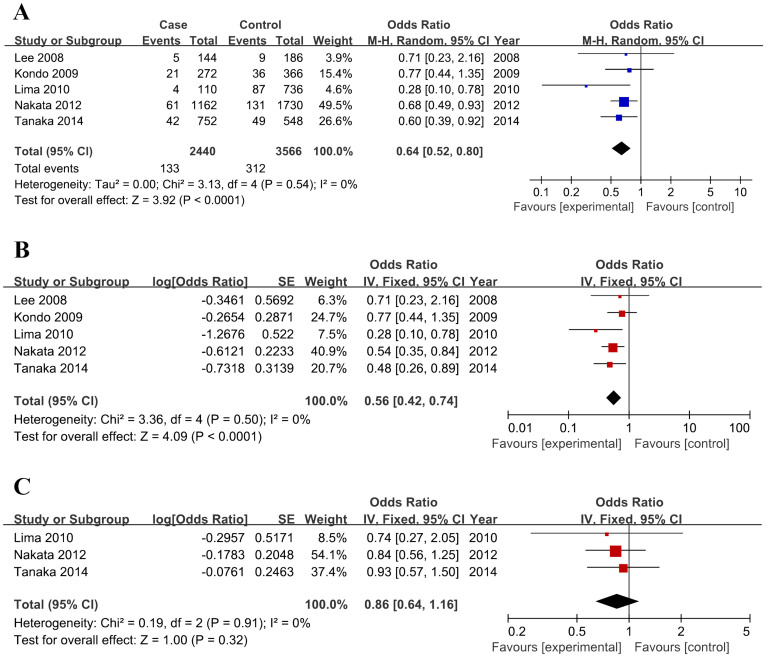
Association between *C2* rs547154 polymorphism and polypoidal choroidal vasculopathy (PCV)/age-related macular degeneration (AMD) risk. Forest plots of *C2* rs547154 polymorphism (T vs G) and PCV risk based on crude (A) and adjusted data (B). Allelic frequencies of *C2* rs547154 polymorphism (T vs G) between PCV and AMD (C).

In the random-effect model, the pooled OR for the risk allele T versus the wild type G was 0.64 (95% CI, 0.52–0.80; *p* < 0.0001) (Figure 2A), pooled OR for genotype TT versus GG was 0.43 (95% CI, 0.15–1.26; *p*, 0.123), pooled OR for genotype TG versus GG was 0.65 (95% CI, 0.52–0.83; *p*, 0.0004), pooled OR for genotype TT + TG versus GG was 0.64 (95% CI, 0.51–0.80; *p*, 0.0002), and pooled OR for genotype TT versus TG + GG was 0.46 (95% CI, 0.16–1.34; *p*, 0.154) (Table 3). No significant heterogeneities exist within all five comparison groups, and results from the fixed-effect model kept consistent with the random-effect model. To minimize the bias, we used adjusted data for better estimation, and the adjusted OR for T versus G was 0.56 (95% CI, 0.42–0.74; *p* < 0.0001) (Figure 2B). No difference in allelic frequency was found between the PCV and AMD groups (OR, 0.86; 95% CI, 0.64–1.16; *p*, 0.32) (Figure 2C). Subgroup analysis based on ethnicity revealed that results from both the Asian and the Caucasian groups kept consistent with overall data in all five tested models, suggesting no existed ethnic divergence (Table 3). Sensitivity analysis was applied to estimate the influence of each study on the pooled OR. No individual study was found to affect the result in all comparison groups, which further proved the robustness of our data. Further, no publication bias was detected via Begg's funnel plot and Egger's test.

## Discussion

Inconsistencies exist in the association between *C2* rs547154 polymorphism and PCV risk, suggesting the necessity to perform an exhaustive review and quantitative analysis on all evidence to determine the effect. In the present study, to assess the association between *C2* rs547154 polymorphism and PCV risk, we reviewed a total of 97 published reports and completed an analysis on 4076 subjects from 5 original studies. Our result suggests that *C2* rs547154 has protective effect on PCV in all populations.

The *C2* locus and the *CFB* locus are located closely together on chromosome 6p^19^. Primary structure of the *C2* gene showed 39% sequence identity with its functionally analogous complement factor B (*CFB*; MIM: 138470)^20^. Component C2, protein encoded by the *C2* gene, shows a wide expression in the neural retina, RPE, and choroid. C2 is part of the classical pathway of the complement system. Upon the binding with component C4b, component C2 is cleaved by activated factor component C1 into two fragments, C2b and C2a^21^. The former fragment, C2b, then combines with the complement factor C4b to generate the C3/C5 convertase. Mutations in *C2* have been implicated in causing complement component 2 deficiency (C2D; MIM: 217000)^22^,^23^, whereas the functional role of *C2* in the etiology of PCV and AMD has not been fully elucidated. Thus, more functional investigations are warranted to give a better insight into the relationship between the *C2* variant and PCV.

To enhance the reliability of our results, we adopted the quality assessment tool recommended by the Newcastle-Ottawa Scale (NOS) for case-control studies. No language limitation was applied. Only studies published in peer-reviewed journals were included. In addition, we also used adjusted data for better estimation of the association. The association between *C2* rs547154 polymorphism and PCV susceptibility has been discussed in a previous report, while they have only included 3 studies and tested the allele model (T versus G)^24^. Several limitations of the present study should be acknowledged. The number of included studies was quite limited, and only one report was on Caucasians. Meanwhile, one study was not included due to the lack of allelic and genotypic data^25^. Therefore, more investigations are warranted to confirm the conclusion, especially in Caucasians and other populations. Despite our effort to narrow down the influence of other risk factors using adjusted data, the number of studies providing adjusted data, especially multivariate-adjusted data, was quite limited. We only conducted subgroup analysis on ethnicity, while the effects of age and gender were not evaluated due to the incomplete data in the control group.

In conclusion, our study suggests that the *C2* rs547154 variant shows a protective effect on the development of PCV. No significant difference was detected between the allelic frequencies in the PCV and the AMD groups. More epidemiological and biological studies are needed to ascertain the association, and to help to understand the molecular basis behind this association.

## Methods

### Literature Searching and Study Eligibility

This meta-analysis was performed according to the PRISMA guidelines^26^. We searched MEDLINE, EMBASE, Web of Science, and the Cochrane Library for all relevant articles using the following three main search terms as indicated previously^11^: (1) (((“Choroid Diseases”[Mesh] AND “Vascular Diseases”[Mesh]) AND polypoidal) OR (polypoidal choroidal vasculopathy OR PCV)) AND ((complement component 2 OR C2 OR) AND (IVS10 OR rs547154))); (2) (“polypoidal choroidal vasculopathy”/exp OR PCV AND (“complement component 2” OR C2) AND (IVS10 OR rs547154)); and (3) ((polypoidal choroidal vasculopathy) OR PCV) AND ((complement component 2 OR C2) AND (IVS10 OR rs547154)). The most recent search was performed on Nov 28, 2014. No language filters were applied. Retrieved records and eligibility status were managed using EndNote X5 software (http://endnote.com/).

Included studies were: (1) studies evaluating the association between *C2* rs547154 polymorphism and PCV risk; (2) genome-wide association studies, or studies of case-control, cohort or population-based epidemiologic design; (3) studies using predefined criteria and procedures for PCV and AMD diagnoses; (4) OR have been reported, or present allelic and genotypic distributions of the *C2* rs547154 variant in PCV cases and control subjects that enable calculations of the outcomes. Alleles included T and G, and genotypes covered TT, TG and GG. We excluded case reports, reviews, conference proceedings, editorials, reports with incomplete data, and only included those with the latest follow-up information for serial publications from the same research team using overlapped subjects. Our study was approved and reviewed by the institutional ethics committee of The First Affiliated Hospital of Nanjing Medical University and adhered to the tenets of the Declaration of Helsinki.

### Data Collection and Quality Evaluation

Two investigators (X.C. and X.K.) independently reviewed all retrieved articles based on title, abstracts, and complete document if necessary. They also extracted data from each study separately with a customized datasheet. All data obtained from the two reviewers were compared. Disagreements were resolved through consensus with a senior reviewer (C.Z.). Data collected included: first author, year of publication, country of sample origin, ethnicity, genotyping method, demographics of subjects, average age and gender ratio of each studied group, allelic and genotypic distributions in cases and controls, adjusted OR and adjusted factors. The NOS (accessed via http://www.ncbi.nlm.nih.gov/books/NBK35156/) was applied for evaluation of the risk of biases for included studies.

### Data Synthesis and Analysis

Hardy-Weinberg equilibrium was calculated using χ^2^ test to compare the genotypic frequencies of the *C2* rs547154 variant among the control subjects. Heterogeneity among studies was assessed using Cochran's Q statistic, and evaluated by the proportion of variation attributable to among-study heterogeneity, *I*^*2*^. Heterogeneity was determined as indicated previously^27^.

The following five ORs and their 95% CI were calculated using both random-effect (the DerSimonian and Laird method)^28^ and fixed-effect model (the Mantel-Haenszel method)^29^: T versus G (allele model), TT versus GG (homozygous model), TG versus GG (heterozygous model), TT + TG versus GG (dominant model), and TT versus TG + GG (recessive model). Subgroup analysis was performed by ethnicity. Sensitivity was used to affirm the results by removing one individual study each time. Begg's funnel plot^30^ and Egger's test^31^ were applied to appraise the publication bias and small-study effects. All analyses were conducted with Review Manager (version 5.2; Cochrane Collaboration, Oxford, UK; http://ims.cochrane.org/revman) and STATA software (version 12.0; StataCorp LP, College Station, TX). Alpha was set to 0.05 for two-sided test.

## Author Contributions

Study design: X.C., K.Z. and C.Z. Collected the samples and performed the experiments: X.C. and X.K. Data interpretation and analysis: X.C. and X.K. Wrote the manuscript: X.C. and C.Z. All authors have read and approved the final manuscript.

## Supplementary Material

Supplementary Information Supplementary Table 1

## Figures and Tables

**Table 1:** Characteristics of Included Studies

Author (Publication Year)	Region (Ethnicity)	Genotyping Method	Total (N)			Average Age (yrs)			Gender Ratio (M/F)		
			PCV	AMD	Control	PCV	AMD	Control	PCV	AMD	Control
Lee et al (2008)^14^	Singapore (Chinese)	Sanger sequencing	72	NA	93	63.80 ± 7.60	NA	67.20 ± 4.60	46/26	NA	40/53
Kondo et al (2009)^15^	Japan (Japanese)	TaqMan	136	NA	183	73.00 ± 6.80	NA	72.00 ± 5.80	105/31	NA	110/73
Lima et al (2010)^16^	USA (Caucasian)	PCR-RFLP/TaqMan	55	368	368	73.00 ± 8.20	NA	NA	32/23	NA	NA
Nakata et al (2012)^17^	Japan (Japanese)	TaqMan	581	455	865	72.59 ± 8.13	75.59 ± 8.60	48.22 ± 16.18	420/161	330/125	431/434
Tanaka et al (2014)^18^	Japan (Japanese)	TaqMan	376	250	274	70.00 ± 8.90	73.60 ± 7.50	72.90 ± 7.40	266/110	187/63	110/164

Abbreviations: RFLP, restriction fragment length polymorphism; PCV: polypoidal choroidal vasculopathy; AMD: age-related macular degeneration; NA: not available; yrs: years; M/F: male/female.

**Table 2:** Genotype Distribution and Allele Frequency of Included Studies

	PCV				Control							
		Genotype				Genotype				Adjusted Results (T vs G)		
Author	(N)	TT/TG/GG	T/G	T (%)	(N)	TT/TG/GG	T/G	T (%)	HWE *p* Value	OR [95% CI]	*p* Value	Adjusted Factors
Lee et al^14^	72	0/5/67	5/139	3.47	93	0/9/84	9/177	4.84	0.624	NA	NA	NA
Kondo et al^15^	136	1/19/116	21/251	7.72	183	2/32/149	36/330	9.84	0.848	NA	NA	NA
Lima et al^16^	55	0/4/51	4/106	3.63	368	5/77/286	87/649	11.82	0.943	NA	NA	NA
Nakata et al^17^	581	2/57/522	61/1101	5.25	865	5/121/739	131/1599	7.57	0.984	0.54 [0.35–0.84]	0.006	Age, Sex
Tanaka et al^18^	376	1/40/335	42/710	5.59	274	4/41/229	49/499	8.94	0.179	0.48 [0.26–0.89]	0.018	Multiple factors†

†Age, Sex, Hypertension, DM, Smoking.

Abbreviations: PCV: polypoidal choroidal vasculopathy; HWE: Hardy–Weinberg equilibrium; NA: not available; OR: odds ratio; CI: confidence interval.

**Table 3:** Meta-analysis for *C2* rs547154 polymorphism and PCV risk

			Sample Size		Random-Effect Model		Fixed-Effect Model		Heterogeneity		
Model	Ethnicity	Studies (n)	Case	Control	OR [95% CI]	*p* Value	OR [95% CI]	*p* Value	*I*^*2*^ (%)	*p* Value	Egger's Test
T vs G (Allele)	Overall	5	2440	3566	0.64 [0.52, 0.80]	<0.0001	0.63 [0.51, 0.78]	<0.0001	0.00	0.536	0.425
	East Asian	4	2330	2830	0.67 [0.53, 0.84]	0.0005	0.67 [0.53, 0.84]	0.0005	0.00	0.925	0.710
	Caucasian	1	110	736	0.28 [0.10, 0.78]	0.015	0.28 [0.10, 0.78]	0.015	NA	NA	NA
TT vs GG (Homozygous)	Overall	4	1028	1419	0.43 [0.15, 1.26]	0.123	0.42 [0.14, 1.21]	0.106	0.00	0.824	0.861
	East Asian	3	977	1128	0.42 [0.13, 1.33]	0.140	0.40 [0.13, 1.25]	0.116	0.00	0.640	0.785
	Caucasian	1	51	291	0.51 [0.03, 9.29]	0.646	0.51 [0.03, 9.29]	0.646	NA	NA	NA
TG vs GG (Heterozygous)	Overall	5	1216	1767	0.65 [0.52, 0.83]	0.0004	0.64 [0.51, 0.81]	0.0002	0.00	0.631	0.424
	East Asian	4	1161	1404	0.68 [0.54, 0.87]	0.002	0.68 [0.54, 0.87]	0.002	0.00	0.985	0.454
	Caucasian	1	55	363	0.29 [0.10, 0.83]	0.021	0.29 [0.10, 0.83]	0.021	NA	NA	NA
TT + TG vs GG (Dominant)	Overall	5	1220	1783	0.64 [0.51, 0.80]	0.0002	0.63 [0.50, 0.79]	<0.0001	0.00	0.570	0.420
	East Asian	4	1165	1415	0.67 [0.53, 0.85]	0.0009	0.67 [0.53, 0.85]	0.0009	0.00	0.968	0.587
	Caucasian	1	55	368	0.27 [0.10, 0.78]	0.015	0.27 [0.10, 0.78]	0.015	NA	NA	NA
TT vs TG + GG (Recessive)	Overall	4	1148	1690	0.46 [0.16, 1.34]	0.154	0.44 [0.15, 1.28]	0.133	0.00	0.820	0.915
	East Asian	3	1093	1322	0.44 [0.14, 1.39]	0.163	0.42 [0.14, 1.31]	0.136	0.00	0.642	0.782
	Caucasian	1	55	368	0.60 [0.03, 10.92]	0.727	0.60 [0.03, 10.92]	0.727	NA	NA	NA

**Abbreviations**: PCV: polypoidal choroidal vasculopathy; OR: odds ratio; CI: confidence interval.
