# Supplementary material for: C2 rs547154 polymorphism and polypoidal choroidal vasculopathy susceptibility: a meta-analysis
Source: Sci Rep. 2015 Mar 3;5:8709. doi: 10.1038/srep08709 (PMC4346790; doi:10.1038/srep08709)
Supplement: Supplementary Information — Supplementary Table 1 [file srep08709-s1.doc]

***C2* rs547154 polymorphism and polypoidal choroidal vasculopathy susceptibility: a meta-analysis**

Xue Chen^1^, Xiaoli Kang^2^, Kanxing Zhao^3^, Chen Zhao^1^

**Supplementary information file**

| **Table S1. Quality Assessment for Included Studies** | | | | | | | | | | | |
| --- | --- | --- | --- | --- | --- | --- | --- | --- | --- | --- | --- |
| **Author** | **Quality Indicators From Newcastle-Ottawa Scale** | | | | | | | | | | |
|  | **Selection** | | | |  | **Comparability** | |  | **Exposure** | | |
|  | **1** | **2** | **3** | **4** |  | **5A** | **5B** |  | **6** | **7** | **8** |
| Lee et al | Yes | Yes | No | Yes |  | Yes | No |  | No | Yes | No |
| Kondo et al | Yes | Yes | No | Yes |  | Yes | No |  | No | Yes | No |
| Lima et al | Yes | Yes | No | Yes |  | Yes | No |  | No | Yes | No |
| Nakata et al | Yes | Yes | No | Yes |  | Yes | No |  | No | Yes | No |
| Tanaka et al | Yes | Yes | NA | Yes |  | Yes | No |  | No | Yes | No |
| Abbreviations: 1: Case is defined with independent validations; 2: Consecutive or obvious representative series of cases; 3: Community controls; 4: Controls are defined as no history of disease; 5A: Study controls for the most important factor; 5B: Study controls for any additional factor; 6: Ascertainment of exposure is secure record; 7: Same method of ascertainment for cases and controls; 8: Same non-response rates for both groups; NA: not available. | | | | | | | | | | | |
